# Global Trends and Research Topics in Gastric Bypass Clinical Trials: A Bibliometric Analysis and Latent Dirichlet Allocation (LDA) Study

**DOI:** 10.1007/s11695-026-08750-x

**Published:** 2026-05-26

**Authors:** Rômulo Ferreira Monteiro, Victor Celestino Pires, Tiago Rafael Onzi, Giovani Firpo Del Duca, Jucemar Benedet

**Affiliations:** 1 Department of Physical Education, Universidade Federal de Santa Catarina, Florianópolis, Brazil; 2 Department of Surgery, Universidade Federal de Santa Catarina, Florianópolis, Brazil

**Keywords:** Gastric bypass, Clinical trial, Bibliometrics, Bariatric surgery, Latent dirichlet allocation, RYGB

## Abstract

**Introduction:**

Gastric bypass (GB) is a widely adopted metabolic bariatric surgical procedure associated with substantial weight loss and significant metabolic benefits. In light of the expanding body of literature in this field, the present study aimed to map and analyze the global scientific output of clinical trials on GB, with emphasis on overall publication trends, the most productive countries and authors, the ten most highly cited articles, and the thematic evolution of research topics.

**Methods:**

The search term “gastric bypass” was used to retrieve publications indexed in the Web of Science, PubMed, and Scopus databases from 2001 to 2024. Bibliometric indicators were integrated with normalization strategies based on population size, the number of bariatric procedures performed, and the prevalence of obesity. Data on authorship, countries, citation counts, and keywords were extracted and analyzed. Topic modeling was conducted using Latent Dirichlet Allocation (LDA).

**Results:**

A total of 1,066 studies were included, demonstrating an average annual growth rate of 10.2%, with a marked expansion observed after 2013. The United States led in absolute publication volume, whereas European countries exhibited higher relative productivity after normalization. The ten most highly cited studies accumulated more than 12,000 citations. Topic modeling identified emerging, stable, and declining thematic domains, with dominant topics primarily focused on diabetes mellitus and showing continued growth.

**Conclusion:**

The United States and Europe dominate scientific output, while several regions remain underrepresented. The most prominent and expanding research themes involve diabetes mellitus, whereas cardiovascular aspects, thromboembolism prophylaxis, hepatic steatosis, gut microbiota, and mental health remain comparatively underexplored in the literature.

**Supplementary Information:**

The online version contains supplementary material available at 10.1007/s11695-026-08750-x.

## Key Points

- The USA leads in absolute output, while Europe leads after normalization.
- Topic modeling identified type 2 diabetes as the predominant research focus.
- Mental health outcomes remain underexplored in the literature.
- Cardiovascular disease and thromboembolism are underrepresented areas.

**Supplementary Information:**

The online version contains supplementary material available at 10.1007/s11695-026-08750-x.

## Introduction

Metabolic bariatric surgery (MBS) has historical records dating back to the 10th century, when King Sancho I of León reportedly underwent a rudimentary intervention involving oral suturing as an early attempt at weight control [1]. In contemporary practice, MBS encompasses a spectrum of gastrointestinal surgical procedures aimed at inducing weight loss in individuals with severe obesity and reducing the risk of associated comorbidities, such as type 2 diabetes mellitus (T2DM) and hypertension. These procedures exert their effects through mechanisms including gastric restriction, alterations in intestinal transit, and hormonal modulation that collectively promote weight loss and metabolic improvement [2].

Obesity is recognized as one of the major public health challenges of the 21 st century [3, 4]. Its global prevalence has more than doubled since 1990, and in 2022 it was estimated that one in eight individuals worldwide was living with obesity. In its most severe forms, class III obesity (body mass index ≥ 40 kg/m²) is projected to affect more than 110 million adults by 2030, underscoring a worsening epidemiological scenario [5, 6]. Within this context, MBS has been established as the reference standard treatment for severe obesity, owing to its effectiveness in achieving sustained weight loss and improving obesity-associated metabolic diseases [7].

Against this backdrop, the number of MBS procedures performed worldwide has increased steadily. In 2014, a total of 100,092 procedures were reported, rising to 598,736 in 2023, reflecting the global expansion of this therapeutic approach [8, 9]. Among the techniques currently available in MBS, GB stands out as one of the oldest, most established, and most widely performed procedures [10], particularly due to its metabolic effects and the high percentage of excess weight loss (%EWL). Studies indicate that patients may achieve up to a 57% reduction in total body weight five years after surgery [11].

In parallel with the rising prevalence of obesity and the increasing surgical volume, scientific output in this field has expanded substantially. A search conducted on September 3, 2025, in the Web of Science (WoS), PubMed, and Scopus databases using the term “gastric bypass” retrieved more than 72,000 publications, underscoring both the magnitude of the field and the challenge of comprehensively mapping its scientific evolution. In this context, narrative and bibliometric reviews are frequently utilized by clinicians, researchers, and the general public as primary sources of up-to-date information [12].

Clinical trials are fundamental to the development of health interventions, providing a rigorous framework for evaluating the efficacy and safety of novel treatments. They encompass investigations ranging from early-phase safety assessments to randomized comparisons between therapeutic strategies. By minimizing bias and generating high-quality evidence, clinical trials establish the evidentiary foundation for informed clinical decision-making and the implementation of effective interventions. As such, they represent a central pillar of evidence-based medicine and the advancement of high-quality healthcare [13, 14].

Several bibliometric studies have characterized the global scientific output on bariatric surgery, identifying a marked increase in publications since the early 2000 s, with GB accounting for a substantial proportion of the research volume [15, 16]. In contrast, sleeve gastrectomy (SG), although currently the most frequently performed procedure, exhibits a lower cumulative number of publications, likely reflecting its more recent adoption as a primary surgical technique [17]. While scientific output specifically focused on SG has been analyzed [18, 19], a clear gap persists in bibliometric studies specifically addressing clinical trials on GB.

In this context, the present study aimed to map the scientific output on GB by analyzing clinical trials indexed in the three largest international databases between 2001 and 2024. The analysis encompassed overall scientific output, the most prolific countries and authors, and the ten most highly cited articles. To further explore the conceptual structure of the field, LDA and bibliometric methods were applied to identify the main research topics and thematic trends, thereby providing a comprehensive and systematized overview of this research area.

## Materials and Methods

### Search Strategy

The present study adopted the framework proposed by Öztürk et al. [20] as a reference for conducting this bibliometric analysis and for guiding the design of studies of this nature.

The search strategy was developed based on terms used in the literature to describe GB procedures and the different types of clinical trials. The search strategy was structured as follows: ((“gastric bypass” OR “roux-en-y gastric bypass” OR “roux en y gastric bypass” OR “RYGB” OR “laparoscopic roux-en-y gastric bypass” OR “LRYGB”) AND (“clinical trial” OR “clinical trials” OR “randomized controlled trial” OR “randomised controlled trial” OR “RCT” OR “interventional study” OR “controlled clinical trial” OR “controlled trial” OR “clinical research” OR “clinical investigation” OR “therapeutic trial” OR “treatment trial”)).

The search was conducted on September 3, 2025, across three international databases. Only documents classified as clinical trials, published in English, and within the period from 2001 to 2024 were included. The complete search strategy is provided in Supplementary Material 1.

### Database Selection

Searches were conducted in the WoS, Scopus, and PubMed databases, selected for their extensive coverage of the international scientific literature and their relevance to biomedical research. WoS and Scopus are widely used in bibliometric studies due to their high-quality indexing, multidisciplinary scope, and availability of essential metadata for this type of analysis. PubMed presents certain limitations, such as the absence of comprehensive citation count data; however, this limitation was partially mitigated through access to OpenCitations (OC), an open science initiative [21].

The retrieved records were exported in different formats according to each database: PubMed records were saved in PubMed format, WoS records in BibTeX format, and Scopus records in CSV format. These files were subsequently imported into the Bibliometrix software (version 4.0) [12], in which data integration, consolidation, and duplicate removal were performed after import. Following this step, the consolidated dataset was converted into a spreadsheet in Microsoft Excel (version 2016) for subsequent data organization and analysis.

### Inclusion and Exclusion Criteria

Clinical trials conducted in patients undergoing GB, specifically Roux-en-Y gastric bypass (RYGB), performed either as a standalone procedure or in combination with other surgical techniques, were considered eligible for inclusion. For the purposes of this study, clinical trials were defined as prospective studies in which a therapeutic intervention was applied to human participants with the aim of evaluating its effects on health outcomes, in accordance with the World Health Organization (WHO) [22].

Study screening was conducted through the assessment of titles and abstracts by two independent authors, evaluating the degree of alignment of each document with the predefined inclusion criteria. Studies involving adult participants (≥ 18 years) were considered eligible, provided that they investigated outcomes related to the proposed surgical intervention. The analysis period comprised publications from 2001 to 2024, and only studies published in English were included to ensure linguistic homogeneity of the textual corpus used in bibliometric analyses and LDA-based topic modeling.

Studies that did not meet the definition of clinical trials, such as observational studies, reviews, letters to the editor, editorials, study protocols, and preclinical research, among others, were excluded. Articles exclusively addressing bariatric techniques other than GB, such as SG, gastric banding, one anastomosis gastric bypass (OAGB), and related procedures, were also excluded. In addition, duplicate records were removed. Full-text assessment was not required, as eligibility could be determined based on the information provided in the title and abstract. The study selection process was illustrated using a PRISMA 2020 (Preferred Reporting Items for Systematic Reviews and Meta-Analyses) flow diagram, depicting the phases of identification, screening, and final inclusion of studies, and adapted to incorporate additional stages of the study, including scope, analyses, and key tools [23].

## Bibliometric and Statistical Analysis Methods

Bibliometric data, including year of publication, authorship, keywords, study location, citation counts, and other relevant metadata, were extracted from publications identified in the selected databases. Results obtained from the different databases were merged using the Bibliometrix software. Subsequently, data cleaning procedures were performed, including duplicate removal, standardization of author names, completion of citation counts for articles retrieved from PubMed through OC as previously described, and other normalizations relevant to the analysis. The data were then organized into a Microsoft Excel spreadsheet to enable subsequent analyses within Bibliometrix. The complete set of metadata is provided in Supplementary Material 2.

Performance analyses were conducted exclusively for the ten most prolific authors in the sample. The following metrics were considered: total number of published articles, total citations, average citations per year, and country, obtained using the Bibliometrix package. Additionally, specific indicators were calculated: Number of Active Years (NAY), defined as the interval between the year of the first and the last publication within the analyzed period, according to the formula NAY = (Year of last publication − Year of first publication) + 1; and Productivity per Active Year (PAY), calculated as the ratio between the total number of publications and the corresponding NAY (PAY = total number of publications/NAY), representing the average annual scientific output during the active period. Authorship position in the publications (e.g., first author or last author) was determined through manual verification of the articles, and this analysis was likewise restricted to the ten most prolific authors. The results were organized and presented in Table 1.

In addition, the ten most cited publications were identified. For these articles, the following information was collected and analyzed: authors, journal of publication, total number of citations, year of publication, average citations per year, and normalized citation counts, with these data obtained using the Bibliometrix package. The journal impact factor (JIF) was retrieved from the Journal Citation Reports (Clarivate Analytics/WoS, 2024), while sample size, intervention duration, and level of evidence, assessed according to the Journal of Bone & Joint Surgery Levels of Evidence scale [24], were determined exclusively for the ten most cited publications and subsequently organized and presented in Table 2.

A total of 47 countries were identified, of which only the ten most prolific were presented in the figure in a horizontal bar format, including the flags and names of each country. Normalization of publication output was performed by dividing the total number of articles by the total population in 2024 (expressed as the absolute number of individuals) and multiplying the result by one million, thereby expressing the indicator as publications per million inhabitants, based on population data obtained from the United Nations (UN) [35]. For bariatric surgery volume, the total number of publications was divided by the total number of bariatric procedures performed in each country (expressed as the absolute number of surgeries), using data obtained from the 8th Global Registry Report (2023) published by the International Federation for the Surgery of Obesity and Metabolic Disorders (IFSO). The resulting value was multiplied by 1,000 and expressed as the number of publications per 1,000 surgeries performed. However, 59.57% of the 47 countries analyzed had unavailable data (not available [N/A]) for bariatric surgery volume, which may limit the validity and interpretability of the normalization process (see Supplementary Material 3). Finally, for obesity prevalence, the population with obesity in each country was estimated by multiplying the total population in 2024 (absolute number of individuals) by the obesity prevalence rate reported by WHO [36], resulting in the estimated number of individuals with obesity (expressed as the absolute number of individuals). The obesity-adjusted index was then calculated by dividing the number of publications by this estimated population and multiplying the result by one million, yielding an indicator expressed as articles per million individuals with obesity. The results were presented as horizontal bar charts (Fig. 3a and d), and the complete normalization data are provided in Supplementary Material 3.

Citation counts were retrieved exclusively from OpenCitations [37] for publications indexed in PubMed (*n* = 88), as PubMed does not provide citation data. For articles indexed in WoS and Scopus, the citation data originally available in these databases were maintained. The extraction of citation data from OC was performed through its public application programming interface (API), using the pandas, requests, and numpy packages implemented in Python (version 3.13) [37]. Citation counts may also be manually verified by consulting the OC website directly.

For scientific mapping, topic modeling was performed. Titles, abstracts, and keywords were extracted, organized in Microsoft Excel, and subsequently preprocessed in the R software, being then combined into a single textual field per document. Preprocessing included conversion to lowercase, removal of punctuation, non-alphabetic characters, and extra whitespace, as well as lemmatization to reduce lexical dispersion. Standard English stopwords and a custom list were also applied to exclude indexing noise, generic methodological terms, and statistical artifacts. Additionally, terminological normalization and merging of relevant multi-word expressions from the biomedical domain were performed, followed by the generation of n-grams to capture frequent terms in the corpus (see Supplementary Material 4).

Topic modeling was conducted using the Latent Dirichlet Allocation method [38], with collapsed Gibbs sampling. During the model selection stage, multiple values of K were estimated, using 500 iterations for each candidate model and 1,000 iterations for the final model, with hyperparameters fixed at α = 0.1 and η = 0.1. The optimal number of topics was determined based on average probabilistic coherence (CalcProbCoherence) and human interpretability [39]. Models with lower K values exhibited thematic overlap, whereas higher values resulted in excessive fragmentation. The model with K = 25 was selected for providing the most coherent thematic organization, with ten representative terms per topic adopted to enhance interpretability. Coherence metrics for the remaining tested models are available in Supplementary Material 5.

Topics were labeled based on the highest-probability terms, prioritizing those most representative of the distribution estimated by the model, with supervised validation performed by a bariatric surgeon. The complete list of ranked terms by topic is provided in Supplementary Material 5. Finally, the absolute and relative frequencies of topics were calculated based on the identification of the dominant topic (highest probability in the LDA θ matrix) for each document.

Following LDA estimation, the topic probability distribution (θ) was obtained for each document. To assess thematic evolution over time, topic proportions were aggregated by year of publication, and the mean annual proportion of each topic was calculated based on document-level probabilities. Subsequently, a simple linear regression model was fitted for each topic, using year of publication as the independent variable and the mean annual topic proportion as the dependent variable. The slope coefficient (β₁) was used to indicate the direction of the temporal trend. A significance level of *p* < 0.01 was adopted to reduce type I error. Topics were then classified as increasing, decreasing, or stable according to the direction and statistical significance of the regression coefficient. For visualization purposes, figures were generated displaying the topics, their respective labels, and the variation in mean annual proportion (%) over the analyzed period.

The temporal trend of publications was evaluated using Kendall’s correlation coefficient (τ), considering years of publication and the annual number of studies. The average annual growth rate was obtained using Bibliometrix.

Descriptive statistical analyses (means, standard deviations, and medians) and bibliometric analyses were performed using R (version 4.4.3) [40] and the bibliometrix package [12].

## Results

**Fig. 1:**
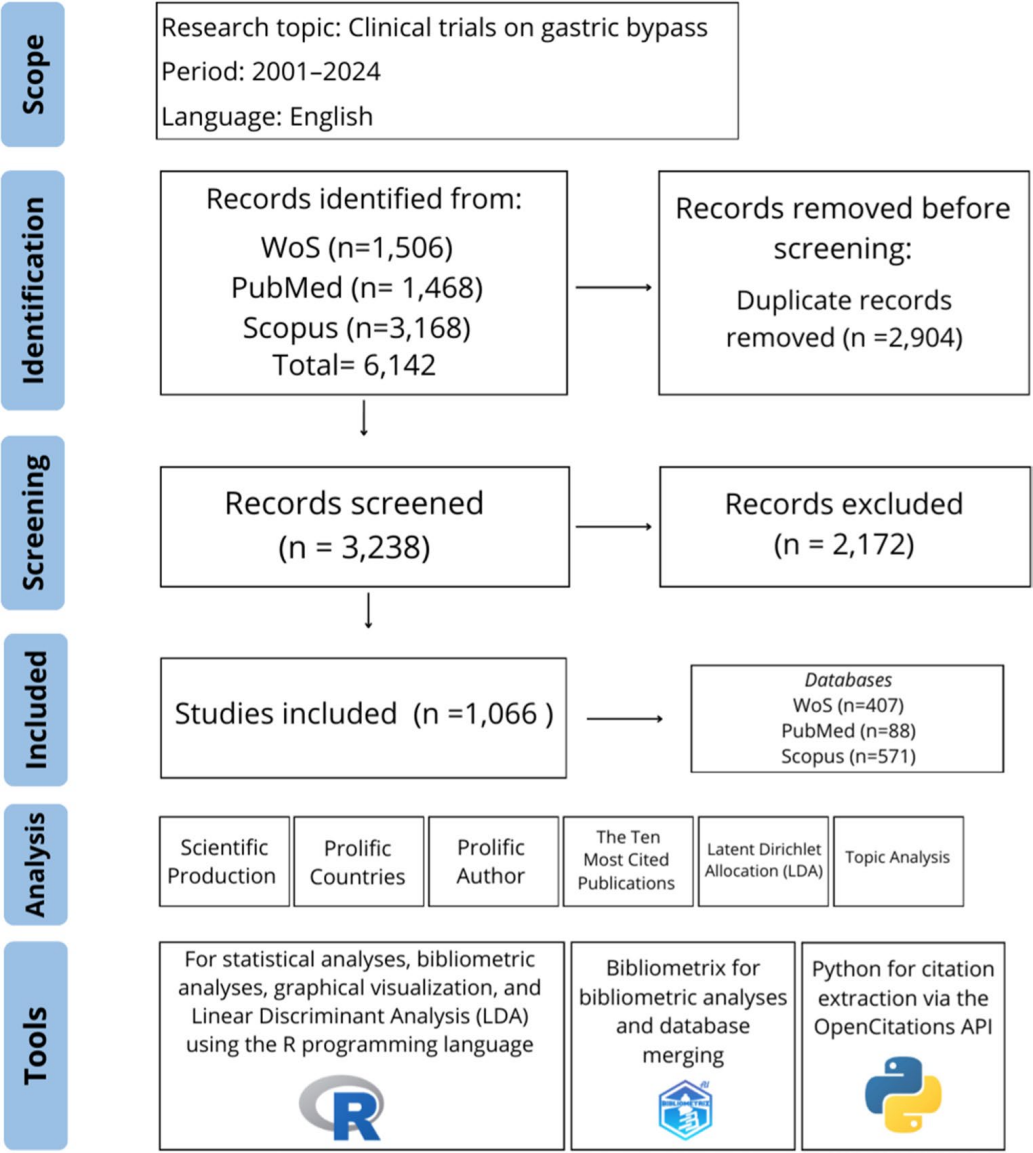
PRISMA flow diagram adapted from the PRISMA 2020 statement

The search strategy identified a total of 6,142 publications, distributed across the WoS (*n* = 1,506), PubMed (*n* = 1,468), and Scopus (*n* = 3,168) databases. After duplicate removal and application of the eligibility criteria, 407 records from WoS, 88 from PubMed, and 571 from Scopus remained, resulting in a final sample of 1,066 publications included in the analysis (Fig. 1).

## Scientific Production

Of the 1,066 articles analyzed, 740 (69.4%) addressed exclusively RYGB, whereas 314 (29.4%) investigated the combination of RYGB and SG, in some cases associated with a third surgical technique. The remaining twelve studies evaluated RYGB in combination with other procedures, such as OAGB, duodenal switch, and adjustable gastric banding.

**Fig. 2:**
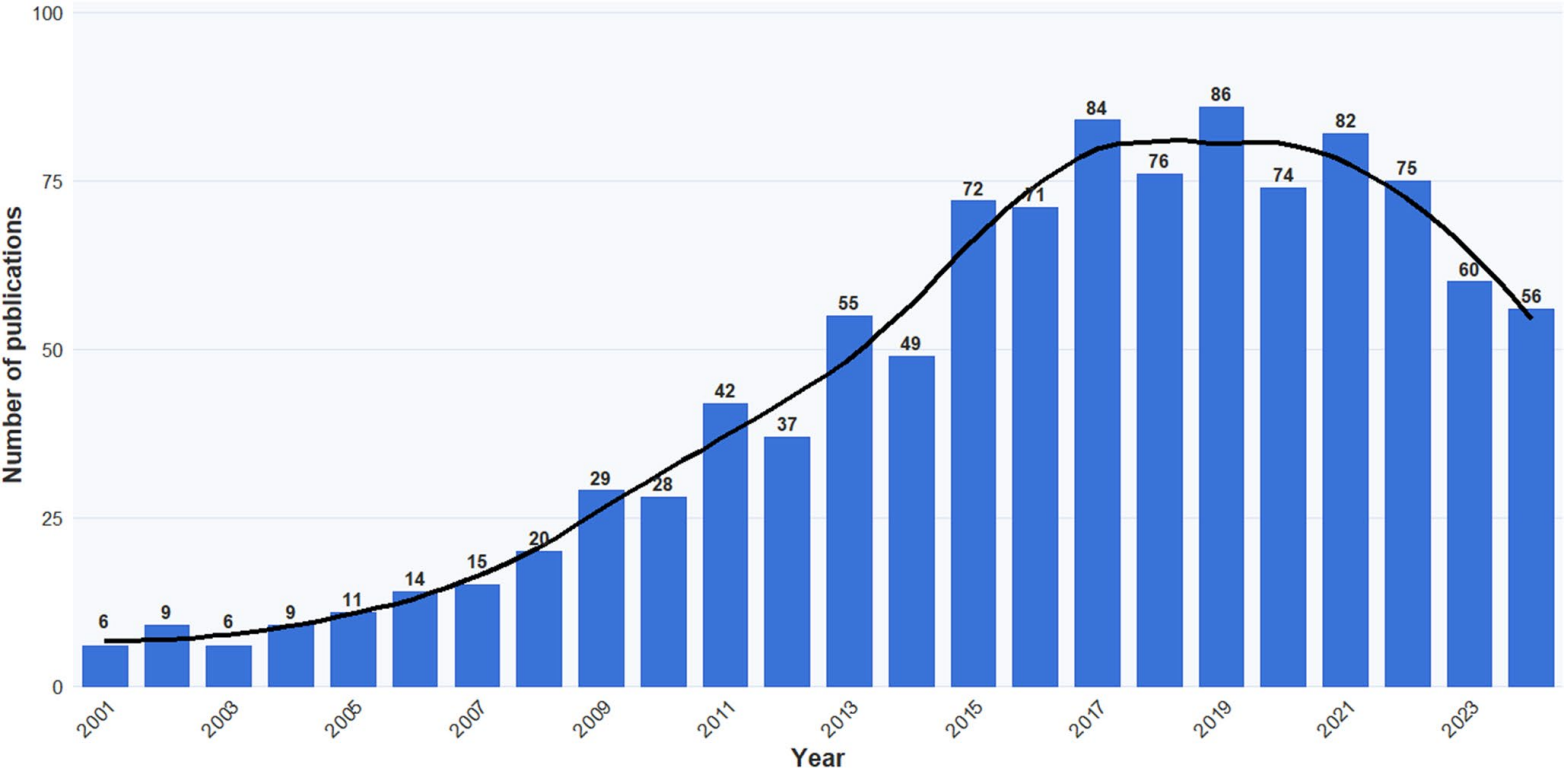
Annual number of publications over the study period, illustrating the yearly distribution and overall trend in scientific output

A statistically significant increasing trend in the number of publications was observed over the analyzed period (τ = 0.764; z = 5.21; *p* < 0.001), with an average annual growth rate of 10.2% (Fig. 2).

An approximate increase of 339.5% in the number of publications was also observed between the first decade (147 articles) and the second decade (646 articles) of the analyzed period. Between 2021 and 2024, a total of 273 publications were recorded, corresponding to 42.3% of the total output of the previous decade.

## Prolific Countries

Contributions to research on clinical trials in GB were identified across 47 countries. The ten most prolific were highlighted for comparative analysis, which considered both absolute publication volume and three normalization indicators: articles per million inhabitants, articles per 1,000 bariatric surgeries, and articles per million individuals with obesity.

**Fig. 3:**
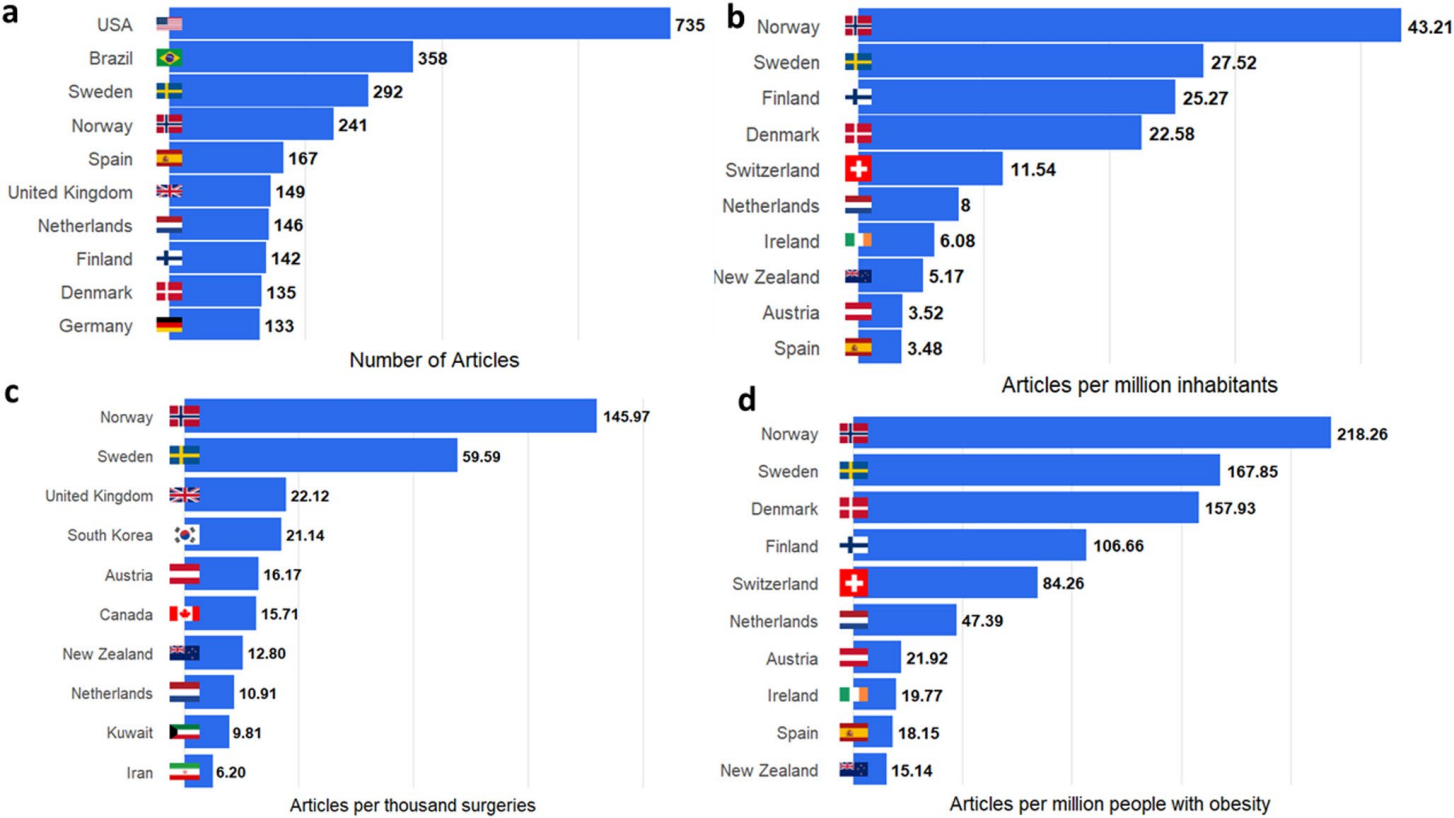
(**a**) The ten most productive countries based on total publication output. (**b**) The ten most productive countries per million inhabitants. (**c**) The ten most productive countries per thousand bariatric surgeries performed. (**d**) The ten most productive countries per million individuals with obesity

Analysis based on absolute scientific output (Fig. 3a) showed that North America and South America account for a substantial share of global production, primarily driven by the high number of publications from the United States and Brazil. In contrast, other South American countries demonstrated limited scientific participation, with publications identified only from Chile, Venezuela, and Argentina, which together did not reach 20 publications within the analyzed corpus (see Supplementary Material 3).

In addition to the United States and Brazil, eight European countries comprised the group of most prolific nations, contributing substantially to the absolute volume of publications. However, when scientific output was normalized by million inhabitants (Fig. 3b), bariatric surgery volume (Fig. 3c), and population of individuals with obesity (Fig. 3d), a shift in the leadership pattern was observed. From this perspective, European countries, particularly those in Northern and Central Europe, emerged as the most prominent, demonstrating high levels of relative scientific productivity. The complete results for other regions are presented in Supplementary Table 3.

## Prolific Author

A total of 5,941 authors contributed to clinical trials on GB within the set of publications included in this study. The mean number of authors per publication was 8.6, and international co-authorship accounted for 9.9% of the studies.

The distribution of productivity showed that approximately 77% (4,590/5,941) of authors published only a single article, whereas only 1.4% (81) were responsible for five or more publications. Table 1 presents the ten most prolific authors.

European authors predominated among the most prolific researchers, and nine of the ten most cited authors demonstrated more than a decade of academic experience, as measured by the number of active years. Nevertheless, despite their extensive experience, mean annual productivity did not exceed four publications per year, as indicated by productivity per active year. A predominance of co-authorship positions was also observed, particularly the last authorship position, which was occupied by the majority of these researchers at least once.

**Table 1:** The ten most prolific authors and their characteristics

Rank	Author	Country	Articles	TC	CY	F- L	NAY-PAY
1	Olbers T	Sweden	32	3,307	183,7	3–9	18–1.89
2	Hjelmesaeth J	Norway	32	966	74.3	2–19	13–2.46
3	Holst J	Denmark	29	2,146	214.6	0–3	10 − 3
4	Le Roux CW	Ireland	27	2,686	206.6	3–9	13 − 2.1
5	Courcoulas A	USA	22	1,695	141.2	6 − 3	12–1.92
6	Hofso D	Norway	21	764	76.4	5–5	10 − 2.1
7	Madsbad S	Denmark	20	1,745	193.8	0–9	9–2.2
8	Sandbu R	Norway	19	553	50.2	0–2	11 − 1.7
9	Kirwan JP	USA	18	6,489	540.7	1–3	12 − 1.5
10	Peltonen M	Finland	16	1,421	142.1	0–1	10 − 1.8

*TC* Total citations

*CY* Citations per year

*F* Number of times the author appeared as first author

*L* Number of times the author appeared as last author

*NAY* Number of active years - Formula: Year of the last publication − Year of the first publication + 1

*PAY* Productivity per active year of publication - Formula: Number of Publications (NP) ÷ Number of Active Years (NAY)

## The Ten Most Cited Publications

Table 2 presents the characteristics of the ten most cited publications. Collectively, these studies accumulated 12,633 citations, with individual counts ranging from 652 to 2,140 citations. This volume corresponds to approximately 19.2% (12,633/65,972) of the total citations across all articles in the corpus, indicating that nearly one-fifth of all citations were concentrated in just ten publications.

The mean number of authors per publication was 12.4 (standard deviation = 3.92), with a median of 10.5. A strong concentration of articles in very high-impact journals was observed, with more than half of these journals presenting a JIF exceeding 78, highlighting the relevance and broad scientific visibility of these publications.

All ten publications presented normalized citation values equal to or greater than 3, indicating that they were cited at least three times above the average of articles published in the same year within the analyzed corpus. Additionally, substantial variability was observed in sample sizes, ranging from 27 to 240 participants, as well as in follow-up periods, with durations varying from a minimum of three months to a maximum of five years.

**Table 2:** Ten most cited articles on gastric bypass and their characteristics

Rank	Authors	Title	Source title	PY	TC	AV	NC	LE	SS	DI
1	Philip R. Schauer et al. [25]	Bariatric Surgery versus Intensive Medical Therapy for Diabetes − 5-Year Outcomes	The New England Journal of Medicine- JIF 2024–78.5	2017	2140	267	27.2	I	150	5
2	Philip R. Schauer et al. [26]	Bariatric Surgery versus Intensive Medical Therapy in Obese Patients with Diabetes	The New England Journal of Medicine- JIF 2024–78.5	2012	1985	152	11.8	I	150	1
3	Geltrude Mingrone, et al. [27]	Bariatric Surgery versus Conventional Medical Therapy for Type 2 Diabetes	The New England Journal of Medicine- JIF 2024–78.5	2012	1601	123	9.5	I	60	2
4	Geltrude Mingrone, et al. [28]	Bariatric metabolic surgery versus conventional medical treatment in obese patients with type 2	The Lancet -JIF 2024 − 88.5	2015	1459	145	18.8	I	60	5
5	Philip R. Schauer, et al. [29]	Bariatric Surgery versus Intensive Medical Therapy for Diabetes − 3-Year Outcomes	The New England Journal of Medicine- JIF 2024–78.5	2014	1374	124	14.5	I	150	3
6	Ralph Peterli, et al. [30]	Effect of Laparoscopic Sleeve Gastrectomy vs. Laparoscopic Roux-en-Y Gastric Bypass on Weight Loss in Patients With Morbid Obesity: The SM-BOSS Randomized Clinical Trial	JAMA Network- JIF 2024–9.7	2018	1008	144	16.7	I	217	5
7	Nguyen NT, et al. [31]	Laparoscopic Versus Open Gastric Bypass: A Randomized Study of Outcomes, Quality of Life, and Costs	Annals of Surgery -JIF − 2024 − 7.5	2001	950	40	3.8	I	155	1
8	Paulina Salminen, et al. [32]	Effect of Laparoscopic Sleeve Gastrectomy vs. Laparoscopic Roux-en-Y Gastric Bypass on Weight Loss at 5 Years Among Patients With Morbid Obesity: The SLEEVEPASS Randomized Clinical Trial	JAMA Network- JIF 2024–9.7	2018	793	113	13.2	I	240	5
9	Sayeed Ikramuddin, et al. [33]	Roux-en-Y gastric bypass vs. intensive medical management for the control of type 2 diabetes, hypertension, and hyperlipidemia: the Diabetes Surgery Study randomized clinical trial	JAMA Network- JIF 2024–9.7	2013	671	56	7.9	I	120	1
10	Ralph Peterli, et al. [34]	Improvement in glucose metabolism after bariatric surgery: comparison of laparoscopic Roux-en-Y gastric bypass and laparoscopic sleeve gastrectomy: a prospective randomized trial	Annals of Surgery -JIF 2024–7.5	2009	652	40	7.0	I	27	0.25

*PY* Publication year, *NC* Normalized citation, *DI *Duration of intervention in years

*TC* Total citation, *LE *Level of evidence, *JIF* Journal Impact Factor

*AV* Average per year, *SS *Sample size

## Coherence Analysis and Model Selection

**Fig. 4:**
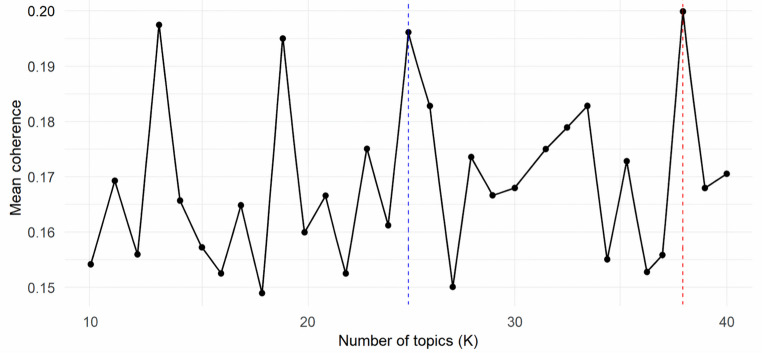
Coherence analysis for optimal topic number selection in the LDA model. The dashed blue line represents the selected model, and the red line indicates the model with the highest coherence score

Coherence analysis indicated that the model with K = 38 (red) achieved the highest mean coherence (0.20); however, it did not demonstrate satisfactory human interpretability. Other coherence values yielded results similar to those observed for the K = 38 model (Fig. 4).

The model with K = 25 (blue) presented a mean coherence of 0.19, a value very close to the maximum observed. Therefore, the model with K = 25 was selected, as it exhibited lower noise levels, improved human interpretability, and a more semantically consistent thematic structure better suited to the analyzed corpus.

## Latent Dirichlet Allocation (LDA) Topic Modeling

With the 25 identified topics, labels were assigned to each cluster, enabling the identification of the main research topics in the literature on clinical trials in GB. Table 3 presents the identified topics, along with the frequency of documents within each topic.

**Table 3:** Topics, keywords, and frequency identified by the LDA model

Topic (Absolute and Relative Frequency%)	keywords
Topic 1- Gut Hormones and Postprandial Glucose Response (75 − 7.0%)	glucagon_like_peptide_1, postprandial, glucose, meal, hormone, test, hormones, responses, plasma, gut
Topic 2 - Glycemic Control and Type 2 Diabetes Remission (42 − 3.9%)	diabetes_mellitus, insulin, dependent, non, type_2_diabetes, type, diabetes, hba1c, hemoglobin, remission
Topic 3 - Postoperative Pain Management and Laparoscopic Recovery (62 − 5.8%)	pain, laparoscopic, recovery, analgesia, nausea, opioid, morphine, scores, stay, analgesic
Topic 4- Thromboembolism Prophylaxis: Efficacy, Safety, and Dosing (14 − 1.3%)	efficacy, risk, safety, dose, sleeve_gastrectomy, daily, prophylaxis, prevention, thromboembolism, second
Topic 5 - Physical Activity and Health Outcomes (50 − 4.6%)	exercise, physical_activity, physical, composition, intervention, health, training, test, program, mass
Topic 6 - Cardiometabolic Risk Factors and Metabolic Profile (21 − 1.9%)	cholesterol, high, lipoprotein, density, glucose, low, circumference, waist, triacylglycerol, metabolic
Topic 7 - Health-Related Quality of Life and Eating Behavior Assessment (51 − 4.7%)	quality_of_life, questionnaire, health, related, symptoms, score, eating, behavior, self, scale
Topic 8 - Dietary Patterns and Nutritional Intake (53 − 4.9%)	diet, intake, low, energy, calorie, dietary, caloric, composition, fat, measured
Topic 9 - Amino Acid and Protein Metabolism with Metabolic Regulation (25 − 2.3%)	acids, metabolism, plasma, acid, metabolic, mass, protein, expression, regulation, metabolites
Topic 10 - Drug administration and pharmacokinetics (37 − 3.4%)	drug, plasma, dose, concentration, oral, area, administration, curve, maximum, single
Topic 11 - Preoperative Predictive Factors (16 − 1.5%)	predictors, preoperative, predictive, predictor, value, high, predicted, center, cohort, presence
Topic 12 - Residual Topic: Non-specific Outcome Reporting Terms (13 − 1.2%)	six, differences, based, evaluated, use, morbidly, assessed, reduced, values, purpose
Topic 13 -Inflammation and Adipose Tissue Biomarkers (45 − 4.2%)	c_reactive_protein, adipose, roux, anastomosis, inflammation, bmi, factor, tissue, protein, inflammatory
Topic 14 -Comparison of Surgical Techniques: Clinical Outcomes (83 − 7.7%)	excess, sleeve_gastrectomy, rouxeny, bmi, laparoscopic, comorbidities, complications, mass, long_term, percentage
Topic 15 -Surgical vs. Medical Treatment for Type 2 Diabetes (57 − 5.3%)	type_2_diabetes, medical, therapy, remission, greater, bmi, intervention, surgical, sleeve_gastrectomy, risk
Topic 16 -Nutritional Deficiencies and Bone Metabolism (52 − 4.8%)	vitamin, supplementation, calcium, deficiency, bone, serum, deficiencies, supplements, nutritional, parathyroid_hormone
Topic 17 - Respiratory Aspects (34 − 3.1%)	pressure, laparoscopic, volume, randomly, preoperative, function, recorded, pulmonary, respiratory, positive
Topic 18 - Glucose–Insulin Metabolism in Type 2 Diabetes (73 − 6.8%)	glucose, insulin_sensitivity, insulin, insulin_resistance, metabolism, type_2_diabetes, tolerance, function, reduction, fasting
Topic 19 - Biliopancreatic diversion and intestinal limb configuration (9-0.8.8%)	biliopancreatic, limb, standard, diversion, biliopancreatic_diversion_duodenal_switch, alimentary, common, long, nutritional, roux
Topic 20 - Cardiovascular Disease and Risk Reduction (25 − 2.3%)	disease, reduction, cardiovascular, heart, hypertension, pressure, therapy, function, sleep, morbidly
Topic 21 - Surgical Complications and Operative Outcomes (111 − 10.4%)	complications, laparoscopic, surgical, operative, laparoscopy, operation, stay, duration, stomach, complication
Topic 22 - Clinical Risk Factors in Surgical Patients (42 − 3.9%)	multicenter, risk, surgical, cohort, care, adjustable_gastric_banding, sex, risk_factor, mass, matched
Topic 23 - Hepatic steatosis and intestinal microbiota (25 − 2.3%)	imaging, liver, fatty, resonance, microbiota, magnetic, function, showed, intestine, gut
Topic 24 - Endoscopic management of weight regain (35 − 3.2%)	endoscopic, endoscopy, pouch, safety, weight_regain, stomach, gastrointestinal, treated, tract, anastomosis
Topic 25 - Clinical Evidence and Trial-Based Comparisons in Sleeve Gastrectomy (16 − 1.5%)	sleeve_gastrectomy, differences, trials, assess, future, three, aimed, among, regarding, evidence

Among the identified topics, five stand out due to their higher representativeness, presented in descending order of frequency: Surgical Complications and Operative Outcomes (topic 21); Comparison of Surgical Techniques: Clinical Outcomes (topic 14); Postprandial Incretin Response and Glycemic Regulation (topic 1); and Postoperative Pain Management and Laparoscopic Recovery (topic 3), collectively accounting for 37.7% of the publications. This prominence indicates a high frequency of occurrence of these themes in the literature over time, suggesting that they represent more consolidated and extensively investigated areas within the field. Type 2 diabetes represents a central focus of the literature, with Topics 2, 15, and 18 collectively accounting for 16% of the total scientific output.

Conversely, the five least represented topics were as follows: Hepatic steatosis and intestinal microbiota (topic 23); Cardiovascular Disease and Risk Reduction (topic 20); Amino Acid and Protein Metabolism with Metabolic Regulation (topic 9); Cardiometabolic Risk Factors and Metabolic Profile (topic 6); and Thromboembolism Prophylaxis: Efficacy, Safety, and Dosing (topic 4). Collectively, they accounted for only 10.1% of the publications in the field.

It is noteworthy that, in addition to the predominant surgical and metabolic domains, topics related to physical activity, quality of life, weight loss and regain, non-communicable chronic diseases, diabetes mellitus, cardiovascular disease, nutritional aspects, and bone health were also identified.

## Topic Trends Analysis

**Fig. 5:**
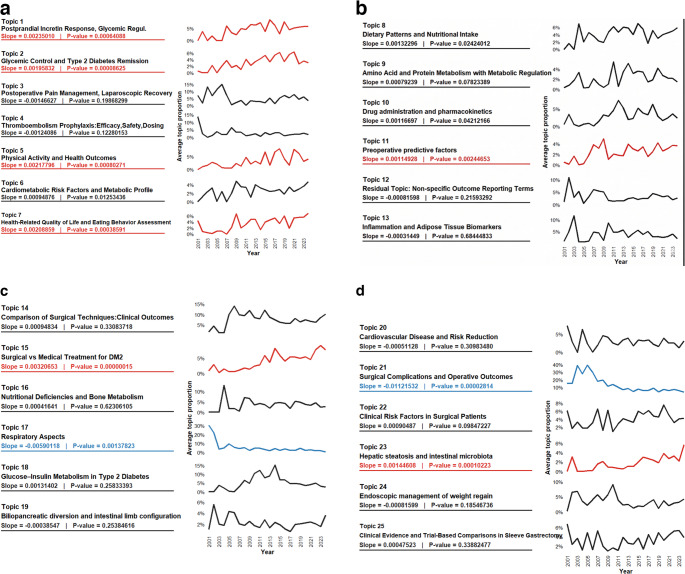
(**a**) Trends of topics 1 to 7 over time: Each panel shows the annual mean proportion of a topic over time. The red line represents significantly rising topics (p < 0.01), the blue line indicates significantly declining topics (p < 0.01), and the black line reflects stable topics without statistical significance. The slope coefficient of the linear regression and its corresponding p-value are presented for each trend. (**b**) Trends of topics 8 to 13 over time. (**c**) Trends of topics 14 to 19 over time. (**d**) Trends of topics 20 to 25 over time

Figure 5a and d present the temporal evolution of topics, their regression coefficients (β), and levels of statistical significance. Topics shown in red indicate growth over time, those in blue indicate decline, and those in black remained stable. Overall, different patterns were observed over time; however, the majority of topics exhibited a stable trend throughout the analyzed period.

The topics Gut Hormones and Postprandial Glucose Response (topic 1); Glycemic Control and Type 2 Diabetes Remission (topic 2); Physical Activity and Health Outcomes (topic 5); Health-Related Quality of Life and Eating Behavior Assessment (topic 7); Surgical vs. Medical Treatment for Type 2 Diabetes (topic 15); and Hepatic steatosis and intestinal microbiota (topic 23) demonstrated a significant increasing trend over the analyzed period, characterizing them as emerging fronts. All these topics showed a mean annual proportion of publications close to 5%.

The topics Respiratory Aspects (topic 17) and Surgical Complications and Operative Outcomes (topic 21) demonstrated a significant declining trend over the analyzed period. Both exhibited a consistent reduction in the mean annual proportion of publications, with higher values in the early years approximately 30–40% followed by a progressive decrease to levels below 10% and 5%, respectively, in more recent years, indicating a substantial decline in their relative representativeness in the literature over time. The topic Respiratory Aspects (topic 17) was widely investigated in the early 2000 s and has shown a sustained decline since 2003. A similar pattern was observed for Surgical Complications and Operative Outcomes (topic 21), although more markedly from 2005 onward.

## Discussion

Metabolic and bariatric surgery is recognized as an effective method for weight loss. Among the available procedures, GB, developed in 1967 by Edward E. Mason and Chikashi Ito [41], remains one of the oldest and most established techniques [42]. Despite the growth of research, there is no comprehensive systematization of clinical trials on this topic. This analysis identified 1,066 clinical trials, mapped the main research topics, identified the most prolific countries, and conducted complementary bibliometric performance analyses within the field.

Scientific output on GB clinical trials showed accelerated growth, with a marked increase from 2013. This trend paralleled the global rise in obesity and related comorbidities, increasing the demand for effective weight-control interventions [4]. Technological advances, including minimally invasive laparoscopy and robotic surgery, improved safety and reduced morbidity and mortality [42–44], further stimulating research activity.

The highest scientific output was observed in 2019; thereafter, it declined steadily. This reduction coincided with the Coronavirus Disease 2019 (COVID-19) pandemic, during which healthcare systems redirected resources and postponed elective procedures such as bariatric surgery, limiting clinical activity and related research [45, 46]. A broader decrease in global biomedical output during the pandemic [45], likely also contributed to this trend.

Regarding scientific output related to specific surgical techniques, the United States previously led research output on laparoscopic sleeve gastrectomy between 1998 and 2019 [18], a pattern that was also observed for GB in the present study. This finding is likely associated with substantial national investment in medical research [46], as well as the high prevalence of obesity in the country, which reached approximately 40.3% of adults between 2021 and 2023 [47, 48].

However, when scientific output was normalized per million inhabitants, per thousand bariatric surgeries performed, and per million individuals with obesity, European countries demonstrated the highest productivity indices, followed by Asian and Oceanian countries. These findings are consistent with previous reports [15, 17, 49] and reflect strong integration between healthcare systems and academic institutions, a high density of research centers, and a consolidated culture of collaboration, which enhance efficiency in knowledge generation.

South American countries, except for Brazil, along with most African nations and parts of the Middle East and Central Asia, remain underrepresented despite increasing obesity rates [50].

Brazil is the only developing country among the ten most prolific nations, ranking second after the United States in absolute output. After normalization, its productivity becomes comparable to that of the United States and corroborates previous findings identifying Brazil as the most productive country in Latin America. This performance may be associated with the marked increase in severe obesity between 2006 and 2021, which has intensified interest in bariatric surgery [51].

The analysis of the ten most prolific authors reinforces the previously observed pattern of global scientific output across countries, highlighting the predominance of researchers affiliated with institutions in the United States and Europe. This concentration reflects persistent geographic disparities in knowledge generation, with limited representation from low- and middle-income regions. Strengthening international collaboration, particularly through partnerships involving underrepresented countries, may constitute a strategic approach to mitigating these disparities and promoting a more balanced distribution of global scientific output.

LDA-based topic modeling identified latent topics encompassing progressively more multidimensional areas.

Topics 2, 15, and 18 primarily focus on T2DM and, collectively, represent the most investigated themes. The association between GB and T2DM remission is evidenced by the recurrence of the term remission within these topics. This finding is consistent with previous studies demonstrating the impact of bariatric surgery on T2DM remission [25, 52, 53].

Despite being well established in the literature, T2DM-related topics have increased significantly over time, indicating sustained research interest. This pattern suggests a continued concentration of the literature on the relationship between diabetes mellitus and bariatric surgery, as evidenced by its recurrence across the most prominent topics identified in the analysis.

Some topics demonstrated low representativeness, each accounting for less than 3% of publications, including cardiovascular disease and risk reduction, thromboembolism prophylaxis (efficacy, safety, and dosing), cardiometabolic risk factors and metabolic profile, amino acid and protein metabolism with metabolic regulation, as well as hepatic steatosis and intestinal microbiota. This distribution indicates that these themes occupy a more limited space within the overall structure of the literature. Notably, hepatic steatosis and intestinal microbiota showed a significant temporal increase, suggesting a growing presence of these topics in recent research and characterizing them as emerging areas in the context of RYGB.

Mental health-related outcomes were not identified in the present analysis. However, existing evidence indicates an increased risk of suicidal ideation and suicide attempts following the procedure compared to non-surgical treatment, with meta-analyses reporting a higher risk of suicide and self-harm behaviors during long-term follow-up. This contrast suggests a limited visibility of these outcomes within the thematic structure identified in the analyzed literature [54, 55].

Finally, bibliometric studies are often limited by reliance on a single database, which may restrict the scope of the analyzed literature [16, 56]. To minimize this limitation, the present study incorporated three databases: WoS, PubMed/MEDLINE, and Scopus [57]. Although PubMed does not provide citation data [58], this limitation was mitigated through the use of open-access citation initiatives, particularly OC [21]. In addition, this study applied topic modeling through LDA, an approach that remains relatively underexplored in bibliometric research within this field.

Nevertheless, some limitations should be acknowledged. Despite efforts to ensure robustness, constraints remain, particularly in the substantiating stage of the sensemaking approach [20, 59], which addresses the dependability, confirmability, and transferability of interpretations. Inconsistencies in metadata across databases may lead to errors in author identification, even after normalization, potentially affecting accuracy. Additionally, the inclusion of only clinical trials may limit the comprehensiveness of the analysis by excluding evidence from observational studies and systematic reviews. In topic modeling, Topic 12 appears to reflect non-specific analytical language rather than a clearly delineated clinical domain, likely representing a residual artifact of the LDA model, a phenomenon commonly observed in LDA-based approaches.

## Conclusion

The global research landscape in the field of GB demonstrates a consistent upward trajectory in scientific output over the analyzed period, despite a slight decline in the most recent years, which may be related to contextual factors. Scientific output remains predominantly concentrated in the United States, with several European nations also holding leading positions. Nevertheless, marked geographic disparities persist, as contributions from various regions with a substantial and growing burden of obesity remain comparatively limited, highlighting ongoing imbalances in the global distribution of research activity.

Research on bariatric surgery remains largely focused on T2DM and metabolic remission. Although its association with improved glycemic control is well established, ongoing studies continue to expand this field. However, future research should move beyond remission and address other clinically relevant diabetes outcomes.

Several clinically important themes remain underrepresented in clinical trials, including cardiovascular aspects, thromboembolism prophylaxis, hepatic steatosis, and gut microbiota. Notably, mental health and psychiatric outcomes did not emerge as structured thematic clusters in our analysis, despite their recognized clinical relevance in the postoperative context.

Overall, the results of this study contribute to the understanding of the trajectory and thematic areas of this research field, particularly within the context of clinical trials throughout the 21 st century.

## Supplementary Information

Below is the link to the electronic supplementary material.

Supplementary File 1 (DOCX 23.7 KB)

Supplementary File 2 (XLSX 3.11 MB)

Supplementary File 3 (DOCX 52.5 KB)

Supplementary File 4 (XLSX 41.2 KB)

Supplementary File 5 (XLSX 21.8 KB)

## Data Availability

The datasets generated and analyzed during the current study are available in Supplementary File 2.
