# Supplementary material for: Global Trends and Research Topics in Gastric Bypass Clinical Trials: A Bibliometric Analysis and Latent Dirichlet Allocation (LDA) Study
Source: Obes Surg. 2026 May 26;36(7):3660–74. doi: 10.1007/s11695-026-08750-x (PMC13323601; doi:10.1007/s11695-026-08750-x)
Supplement: Supplementary file 1 — (DOCX 23.7 KB) [file 11695_2026_8750_MOESM1_ESM.docx]

**Supplementary Material 1 – Search Strategy**

The literature search strategies were developed a priori and consistently applied across all selected databases. Searches were conducted for studies published between 2001 and 2024. All queries were performed in the title, abstract, and keyword fields, when available, to maximize retrieval sensitivity. The search strategy combined terms related to gastric bypass procedures with those associated with clinical trial designs.

**Web of Science**

Search date: 2025-03-09

Coverage: 2001–2024

Fields Searched: Title, Abstract, Keywords

Language: English

Results:1,506

Search Strategy:

TS=(

("gastric bypass"

OR "roux-en-y gastric bypass"

OR "RYGB"

OR "Laparoscopic Roux-en-Y gastric bypass"

OR "LRYGB")

AND

("clinical trial"

OR "clinical trials"

OR "randomized controlled trial"

OR "randomised controlled trial"

OR "RCT"

OR "interventional study"

OR "controlled clinical trial"

OR "controlled trial"

OR "clinical research"

OR "clinical investigation"

OR "therapeutic trial"

OR "treatment trial")

)

**PubMed/MEDLINE**

Search date: 2025-03-09

Coverage: 2001–2024

Fields Searched: Title/Abstract ([tiab]) and MeSH terms

Language: English

Results: 1,468

Search Strategy:

(

"Gastric Bypass"[Mesh]

OR "gastric bypass"[tiab]

OR "roux-en-y gastric bypass"[tiab]

OR "RYGB"[tiab]

OR "Laparoscopic Roux-en-Y gastric bypass"[tiab]

OR "LRYGB"[tiab]

)

AND

(

"Clinical Trial"[Publication Type]

OR "Randomized Controlled Trial"[Publication Type]

OR "Controlled Clinical Trial"[Publication Type]

OR "clinical trial"[tiab]

OR "clinical trials"[tiab]

OR "randomized controlled trial"[tiab]

OR "randomised controlled trial"[tiab]

OR "RCT"[tiab]

OR "interventional study"[tiab]

OR "controlled clinical trial"[tiab]

OR "controlled trial"[tiab]

OR "clinical research"[tiab]

OR "clinical investigation"[tiab]

OR "therapeutic trial"[tiab]

OR "treatment trial"[tiab]

)

**Scopus**

Search date: 2025-03-09

Coverage: 2001–2024

Fields Searched: Title, Abstract, Keywords

Language: English

Results: 3,168

Search Strategy:

TITLE-ABS-KEY(

(

"gastric bypass"

OR "roux-en-y gastric bypass"

OR "roux en y gastric bypass"

OR "RYGB"

OR "laparoscopic roux-en-y gastric bypass"

OR "LRYGB"

)

AND

(

"clinical trial"

OR "clinical trials"

OR "randomized controlled trial"

OR "randomised controlled trial"

OR "RCT"

OR "interventional study"

OR "controlled clinical trial"

OR "controlled trial"

OR "clinical research"

OR "clinical investigation"

OR "therapeutic trial"

OR "treatment trial"

)

)
