# Supplementary material for: Global Trends and Research Topics in Gastric Bypass Clinical Trials: A Bibliometric Analysis and Latent Dirichlet Allocation (LDA) Study
Source: Obes Surg. 2026 May 26;36(7):3660–74. doi: 10.1007/s11695-026-08750-x (PMC13323601; doi:10.1007/s11695-026-08750-x)
Supplement: Supplementary file 3 — (DOCX 52.5 KB) [file 11695_2026_8750_MOESM3_ESM.docx]

| **Country**  **Number of articles:** Total number of articles retrieved from the database search.  **Articles per million inhabitants:** Publication output was normalized by dividing the total number of articles by the total population in 2024 and multiplying the result by one million. The indicator was expressed as articles per million inhabitants. Population data were obtained from the United Nations (UN).  **Articles per 1,000 surgeries:** For normalization, the total number of articles was divided by the total number of bariatric surgeries performed in each country, using data from the 8th Global Registry Report (2023) published by the International Federation for the Surgery of Obesity and Metabolic Disorders (IFSO). The result was multiplied by 1,000 and expressed as the number of publications per 1,000 surgeries performed.  **Articles per million people with obesity:** First, the population with obesity was estimated by multiplying the total population for 2024 (United Nations data) by the obesity prevalence rate for 2022 reported by the World Health Organization (WHO), resulting in the estimated number of individuals with obesity. The obesity-adjusted index was then calculated by dividing the number of publications by this estimated population and multiplying the result by one million, yielding an indicator expressed as articles per million individuals with obesity.  Supplementary Material 3. This material presents the complete results of the normalization process of the number of scientific articles by population, per million individuals with obesity, and by surgical volume for the countries analyzed. A brief description of each indicator is provided below. When data were unavailable, the values were classified as “N/A” (Not Available).  **Supplementary Material 3 – Normalization** | **Number articles** | **Articles per milion inhabitants** | **Articles per Thousand surgeries** | **Articles per million people with obesity** |
| --- | --- | --- | --- | --- |
| USA | 735 | 2.12 | 3.18 | 4.95 |
| BRAZIL | 358 | 1.68 | 5.07 | 5.86 |
| SWEDEN | 292 | 27.52 | 59.59 | 167.85 |
| NORWAY | 241 | 43.21 | 145.97 | 218.26 |
| SPAIN | 167 | 3.48 | N/A | 18.15 |
| NETHERLANDS | 146 | 8.00 | 10.91 | 47.39 |
| UNITED KINGDOM | 149 | 2.15 | 22.12 | 7.50 |
| GERMANY | 133 | 1.57 | N/A | 6.49 |
| FINLAND | 142 | 25.27 | N/A | 106.66 |
| DENMARK | 135 | 22.58 | N/A | 157.93 |
| SWITZERLAND | 103 | 11.54 | N/A | 84.26 |
| CHINA | 95 | 0.06 | 3.15 | 0.81 |
| FRANCE | 92 | 1.38 | 2.36 | 12.68 |
| ITALY | 84 | 1.41 | 5.28 | 6.55 |
| IRAN | 42 | 0.45 | 6.20 | 1.81 |
| AUSTRIA | 34 | 3.52 | 16.17 | 21.92 |
| EGYPT | 34 | 0.29 | N/A | 0.67 |
| BELGIUM | 34 | 2.89 | N/A | 13.16 |
| CANADA | 34 | 0.85 | 15.71 | 3.13 |
| IRELAND | 32 | 6.08 | N/A | 19.77 |
| PORTUGAL | 31 | 2.97 | N/A | 10.97 |
| SOUTH KOREA | 31 | 0.59 | 21.14 | 8.94 |
| AUSTRALIA | 24 | 0.89 | 1.18 | 2.82 |
| NEW ZEALAND | 27 | 5.17 | 12.80 | 15.14 |
| POLAND | 25 | 0.64 | N/A | 2.06 |
| GREECE | 23 | 2.28 | N/A | 6.79 |
| MEXICO | 20 | 0.15 | 2.83 | 0.42 |
| INDIA | 19 | 0.01 | N/A | 0.18 |
| ISRAEL | 18 | 1.91 | 2.71 | 8.19 |
| TURKEY | 17 | N/A | N/A | N/A |
| KAZAKHSTAN | 14 | 0.67 | N/A | 3.52 |
| CHILE | 12 | 0.60 | 0.68 | 1.53 |
| KUWAIT | 11 | 2.22 | 9.81 | 4.91 |
| THAILAND | 11 | 0.15 | N/A | 1.05 |
| SINGAPORE | 9 | 1.54 | N/A | 11.43 |
| SAUDI ARABIA | 8 | 0.23 | N/A | 0.57 |
| JAPAN | 5 | 0.04 | N/A | 0.82 |
| SLOVENIA | 5 | 2.35 | N/A | 10.63 |
| VENEZUELA | 3 | 0.10 | 2.02 | 0.46 |
| ARGENTINA | 2 | 0.04 | N/A | 0.12 |
| BAHRAIN | 2 | 1.24 | N/A | 3.34 |
| NIGERIA | 2 | 0.00 | N/A | 0.07 |
| OMAN | 2 | 0.37 | N/A | 1.25 |
| UNITED ARAB EMIRATES | 2 | 0.18 | N/A | 0.57 |
| JORDAN | 1 | 0.08 | N/A | 0.24 |
| LEBANON | 1 | 0.17 | N/A | 0.55 |
| LITHUANIA | 1 | 0.34 | N/A | 1.12 |
